# Boiling Histotripsy-induced Partial Mechanical Ablation Modulates Tumour Microenvironment by Promoting Immunogenic Cell Death of Cancers

**DOI:** 10.1038/s41598-019-45542-z

**Published:** 2019-06-21

**Authors:** Ki Joo Pahk, Cheol-Hee Shin, In Yeong Bae, Yoosoo Yang, Sang-Heon Kim, Kisoo Pahk, Hyungmin Kim, Seung Ja Oh

**Affiliations:** 1 Center for Bionics, Biomedical Research Institute, Korea Institute of Science and Technology (KIST), Seoul, 02792 Republic of Korea; 2 Center for Biomaterials, Biomedical Research Institute, Korea Institute of Science and Technology (KIST), Seoul, 02792 Republic of Korea; 3 Center for Theragnosis, Biomedical Research Institute, Korea Institute of Science and Technology (KIST), Seoul, 02792 Republic of Korea; 4 Institute for Inflammation Control, Korea University, Seoul, 02841 Republic of Korea; 5 Department of Nuclear Medicine, Korea University Anam Hospital, Seoul, 02841 Republic of Korea

**Keywords:** Biomedical engineering, Breast cancer, Cell death and immune response

## Abstract

Boiling histotripsy is a promising non-invasive High-Intensity Focused Ultrasound (HIFU) technique that employs HIFU mechanical effects to fractionate solid tumours without causing any significant thermal damage. It has been suggested that boiling histotripsy may induce a strong immune response due to the absence of denatured antigenic protein at the HIFU focus. The underlying immunological mechanisms of this technique are, however, poorly understood. In this study, we demonstrated the feasibility of using boiling histotripsy to mechanically fractionate human breast adenocarcinoma cells (MDA-MB-231) and the potential immunological effects induced by boiling histotripsy, for the first time. Our results showed that mechanical stresses produced by boiling histotripsy promote immunogenic cell death of cancer cells via TNF-induced necrosis signaling pathway. This immunogenic cell death significantly increases secretions of damage-associated molecular patterns (CRT, HSP70, HMGB-1), pro-inflammatory cytokines (IFN-γ, IL-1α, IL-1β, IL-18) and chemokines (IL-8) which are related to M1 macrophage activation. Furthermore, the levels of these signaling proteins increase with the degree of mechanical damage induced by boiling histotripsy. Together, the results presented can suggest that boiling histotripsy could be a potential therapeutic approach for not only mechanically destroying solid tumours (e.g., breast cancer) but also promoting immunogenic cell death via TNF-induced necrosis to trigger antitumour immunity.

## Introduction

High-Intensity Focused Ultrasound (HIFU) is a promising non-invasive ultrasonic technique which has been employed to thermally necrose solid tumours without disruption of surrounding tissue^1–3^. This ultrasonic technique essentially involves focusing an ultrasound beam and delivering sufficient amount of acoustic energy into a small region of interest within the body. Most HIFU clinical applications currently rely on a thermal ablative effect^4^. HIFU thermal ablation has received FDA (U.S. Food and Drug Administration) approval for the treatment of uterine fibroids, pain palliation of bone metastases, benign prostatic hyperplasia, prostate cancer and essential tremor^5^. Another biological effect, in addition to the generation of heat, is HIFU-induced mechanical damage mainly due to acoustic cavitation^6^. Recent studies have shown the feasibility of using HIFU to mechanically fractionate soft tissue at the HIFU focus in a controlled manner without inducing coagulative thermal damage. This technique is known as mechanical tissue fractionation or boiling histotripsy (BH). Animal studies have demonstrated that boiling histotripsy can produce well defined mechanically fractionated lesions in kidney, heart and liver which are sharply demarcated between treated and untreated regions without any sign of thermal damage^7–11^.

Breast cancer, among other solid tumours, is particularly well-suited for HIFU therapy because the breast has an excellent acoustic window for ultrasound wave propagation^12^. According to recent clinical guidelines, the current mainstay treatment of breast cancer involves surgical resection with or without radiotherapy, systemic chemotherapy, endocrine therapy, and/or anti-human epidermal growth factor receptor 2 (HER2) therapy^13,14^. Whilst the oncological safety of breast surgery is achieved with current surgical techniques, the surgical procedures involved can, however, lead to certain complications such as bleeding and infections^12^. Furthermore, poor cosmetic outcomes are usually accompanied even after breast-conserving surgery, which removes part of the breast tissue as opposed to the entire breast, is performed. There is, therefore, a high clinical need for the development of minimally invasive or completely non-invasive ablative techniques for the treatment of breast cancer, as an alternative or in addition to current surgical techniques.

In recent years, HIFU has gained significant interests in the field of immunotherapy. A number of clinical and preclinical studies have shown and suggested that HIFU treatment can lead to the development of an active immune response through increases in the levels of cytotoxic T cell activity^15^ and dendritic cell (DC) activation^16,17^. Whilst most studies have shown the potential immunological effects of HIFU thermal ablation, some studies suggest that HIFU-induced mechanical effects may even induce a stronger immune response. This is likely to be due to the absence of denatured antigenic protein at the HIFU focus *in situ* which can further enhance immune reaction^16,18,19^. Schade *et al*.^20^ recently found that boiling histotripsy can significantly increase anti-tumour immunity through acute inflammatory and immunologic responses to mechanical ablation of renal cell carcinoma *in vivo*. Though they clearly demonstrated the high potential of boiling histotripsy for triggering an immune response for the first time, the mechanisms behind the enhancement of antitumour activities following boiling histotripsy treatment are still poorly understood, particularly intracellular signalling cascades that can eventually lead to the generation of pro-inflammatory tumour microenvironment.

To that end, in this study, we aim to (a) investigate the feasibility of employing boiling histotripsy in the treatment of human breast cancer for the first time; (b) examine the relationship between the degree of mechanical damage induced by boiling histotripsy and the level of immune activity, and (c) finally to provide a better understanding of the effects of boiling histotripsy on immune response. *In vitro* experiments with human breast adenocarcinoma cells (MDA-MB-231) are performed in the present study.

## Materials and Methods

### HIFU experimental setup

**Figure 1:**
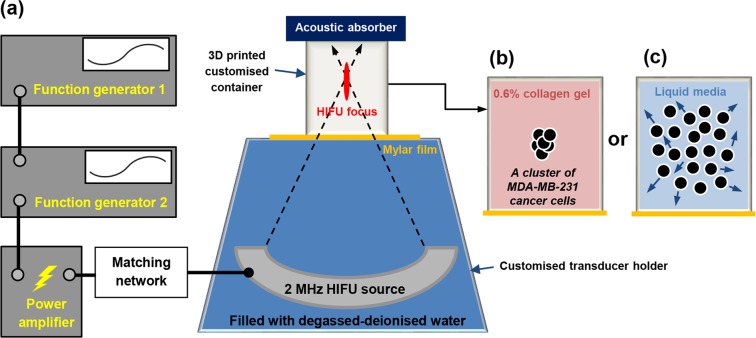
(**a**) HIFU experimental setup used in the present *in vitro* study with a cluster of MDA-MB-231 breast cancer cells (●) (**b**) embedded in 0.6% collagen gel or (**c**) in liquid media.

A schematic diagram of the experimental setup used in the present study is shown in Fig. 1. Two different sets of *in vitro* HIFU experiments were performed to investigate the potential immunological effects of boiling histotripsy. A number of clusters of MDA-MB-231 cells (human breast adenocarcinoma) in the form of spheroids embedded in 0.6% collagen gel in a cylindrical container were exposed to the field of a 2.0 MHz single element bowl-shaped HIFU transducer (H148, Sonic Concepts, Bothell, WA, USA). This HIFU transducer with an aperture size of 64 mm and a radius of curvature of 63.2 mm was driven by two function generators (33220A, Agilent, CA, USA) via a linear power amplifier (1040 L, ENI, NY, USA).

During the experiments, the 2.0 MHz HIFU source coupled with a customised transducer holder filled with degassed and deionised water was initially placed on a lab bench facing upward. The cylindrical container (outer diameter OD of 20 mm, inner diameter ID of 16 mm and height H of 16 mm) containing 0.6% collagen gel, which was 3D printed using PLA (Polylactic acid) on a LulzBot TAZ 5 printer (LulzBot, CO, USA) at a layer resolution of 0.2 mm, was then positioned on top of the transducer holder. The transducer holder and the container were placed coaxially. For coupling purpose, a 12 *μ*m thick acoustically transparent polyethylene (Mylar) film (PMX 980, HiFi Industrial Film, Stevenage, UK) was attached to the bottom surface of the container. Lastly, an acoustic absorber (AptFlex F28, Precision Acoustics Ltd, Dorset, UK) was placed opposite end to the HIFU source to minimise potential ultrasonic reflections (see Fig. 1). The HIFU focus was 5 mm above the Mylar film. After boiling histotripsy exposure, MDA-MB-231 cells embedded in the collagen gel were collected for morphological analysis under a confocal microscope.

An additional set of HIFU experiments was carried out (see Fig. 1c), this time using MDA-MB-231 cells cultured in the liquid media at a density of 2 × 10^5^ cells/3 mL, in order to investigate the types of cell death (apoptosis, autophagy or necrosis) and secreted antigenic factors resulting from boiling histotripsy exposure. Boiling histotripsy-treated human cancer cells were also co-cultured with human monocytes (THP-1 cells) or human M2 macrophages to examine macrophage polarisation (M1 or M2 differentiation) and reprogramming. All analyses were carried out using cell death pathway, proteome cytokine, and quantitative polymerase chain reaction (qPCR) arrays.

### HIFU exposure conditions

During the HIFU experiments, a 10 ms-long HIFU pulse with an electrical power *P*_elect_ of 200 W supplied to the HIFU transducer was used to produce a boiling histotripsy lesion in the collagen gel. The pulse repetition frequency (PRF) of 1 Hz and the duty cycle of 1% were kept constant whilst varying the number of HIFU pulses of 5, 25, 100 or 200.

### Acoustic characterisation of the HIFU transducer

**Figure 2:**
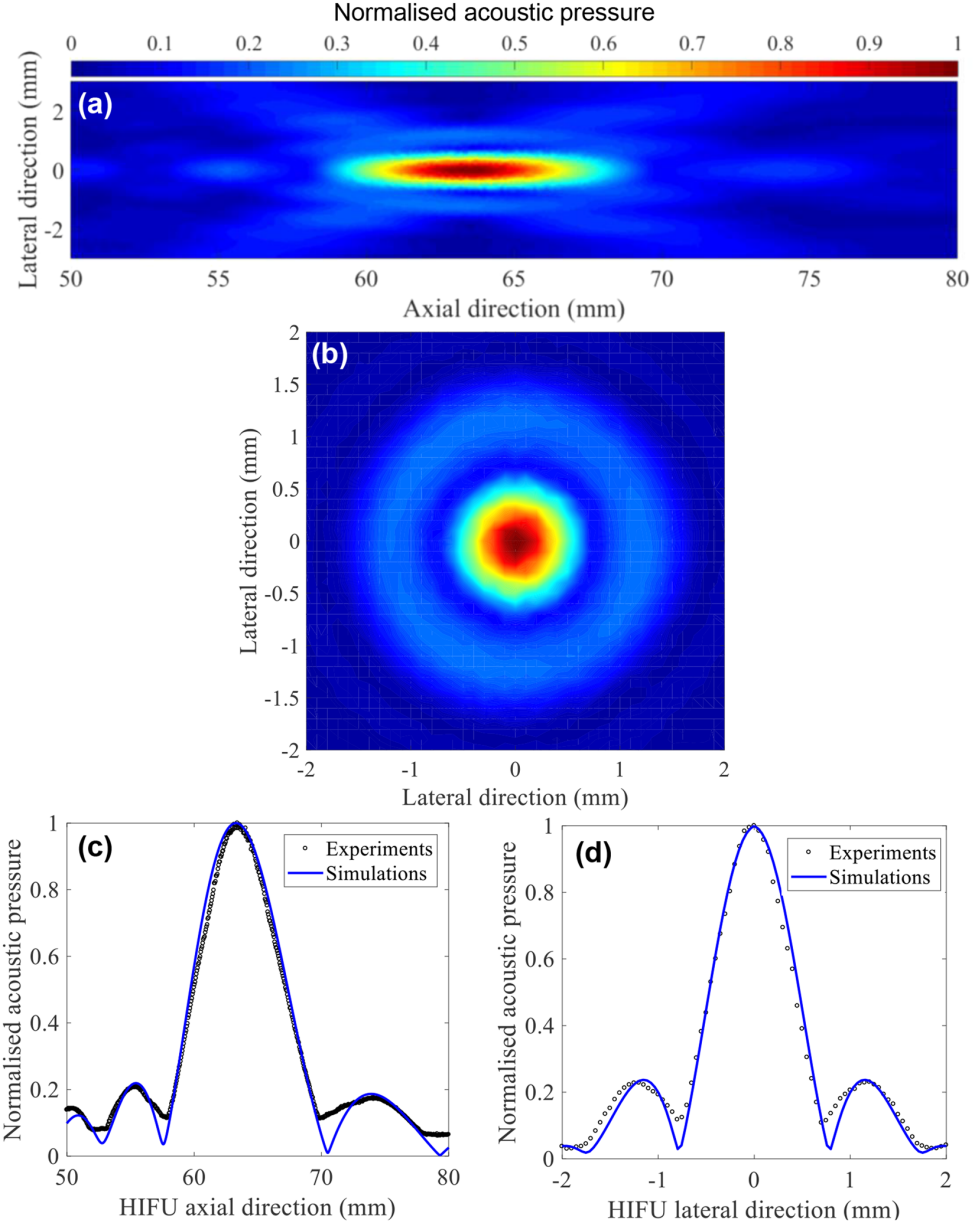
Acoustic characterisation of the 2.0 MHz HIFU transducer (H148, Sonic Concepts, USA) employed. Experimentally measured two-dimensional HIFU focal pressure beam profiles along (**a**) the axial and (**b**) the lateral directions. Measurements were taken in degassed and deionised water. Comparison of one dimensional (**c**) axial and (**d**) lateral focal pressure fields measured in water by the needle hydrophone and calculated with the linearised Khokhlov-Zabolotskaya-Kuznetsov (KZK) wave equation. The lateral and axial full width half maximum (FWHM) dimensions of the transducer used were 0.89 mm and 7.25 mm, respectively.

The HIFU transducer used in the present study was experimentally characterised in degassed and deionised water under linear propagation conditions with a calibrated needle hydrophone (HNP-0400, ONDA, Sunnyvale, CA, USA) and an acoustic field scanning system (AIMS III, ONDA) at a spatial step size of 0.05 mm. The measured HIFU focal pressure beam profiles along the axial and lateral directions are plotted in Fig. 2. It was determined that the HIFU focal distance was 63.4 mm (from the surface of the transducer) and the lateral and axial full width half maximum (FWHM) pressure dimensions were 0.89 mm and 7.25 mm, respectively.

**Figure 3:**
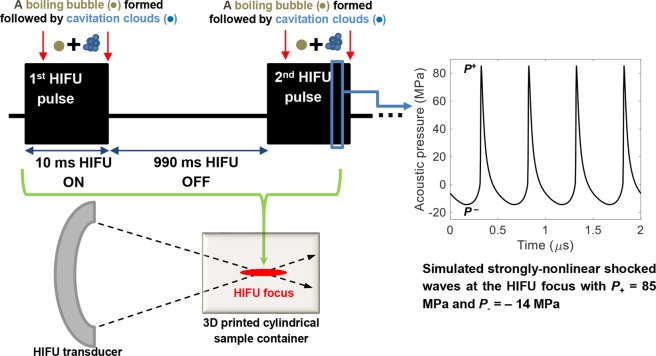
HIFU pulsing protocol used in the present study. 10 ms long HIFU pulse, 1 Hz pulse repetition frequency, 1% duty cycle and the number of HIFU pulses of 5, 25, 50, 100 or 200 were employed. Simulated peak positive *P*_+_ and negative *P*_*−*_ pressures at the HIFU focus were 85 and −14 MPa *in situ*.

Figure 3 shows the HIFU pulsing protocol used in the present study for inducing boiling histotripsy lesions in the collagen gel. With *P*_elect_ of 200 W supplied to the HIFU transducer, strongly-nonlinear shocked waves with *P*_+_ of 85 MPa and *P*_−_ of – 14 MPa were to be produced at the HIFU focus *in situ*, which were obtained by numerically solving the Khokhlov-Zabolotskaya-Kuznetsov (KZK) nonlinear wave equation using the HIFU Simulator v1.2^21^. The ultrasound exposure conditions employed in this study were well within the range of required exposure conditions reported by other boiling histotripsy studies^7,10,11,22^. Therefore, boiling vapour bubbles and cavitation clouds, which occur during the course of boiling histotripsy exposure and are responsible for mechanical tissue fractionation^7,22–24^, were expected to be formed within the HIFU focal zone in the collagen gel during each HIFU pulse. Acoustic properties of the collagen gel used in the KZK simulation are shown in Supplementary Table S1.

### Human cell lines and reagents

Human cancer cell lines MDA-MB-231 (HTB-26™) and human monocytes cell lines THP-1 (TIB-202™) cells were purchased from American Type Culture Collection (Rockville, MD, USA), and maintained in Roswell Park Memorial Institute (RPMI) medium supplemented with 10% FBS from Gibco (Carlsbad, CA, USA) and antibiotics (100 U/ml penicillin, 100 μg/ml.

streptomycin) at 37 °C in a humidified atmosphere with 5% CO2. All these cell lines were authenticated by the ATCC (American Type Culture Collection) and were consistent with their presumptive identity. Cisplatin and doxorubicin were purchased from Sigma Aldrich (Saint Louis, MO, USA).

### 3D cancer cell spheroid formation

MDA-MB-231 cells were plated on the poly(2-hydroxyethyl methacrylate) (pHEMA) coated U-bottom plates at a density of 2 × 10^5^ cells per well with 50 μL RPMI-1640 growth media. After centrifugation, 50 μL of 10% Matrigel solution (Corning, Cat#354234) was gently added to the plate. This was centrifuged one more time and was finally incubated for 72 hours at 37 °C in a humidified atmosphere with 5% CO_2_.

### Embedding 3D cancer spheroids into collagen gel at the HIFU focus

In our experiments, 3D breast cancer cell spheroids embedded in 0.6% collagen gel were placed at the location of the HIFU focus in the 3D printed cylindrical container (OD = 20 mm, ID = 16 mm and H = 16 mm). Initially, 1 mL of 0.6% collagen solution (Corning, Cat#354236) was poured into the container (Supplementary Fig. S1a). Following this, a 3D-printed T-shaped bar was immediately placed in the collagen solution, where the tip of the bar with a diameter of 8 mm indicated the location of the HIFU focus in the container (Supplementary Fig. S1b). The collagen solution was incubated for 10 mins at 37 °C to gelation. The bar was then gently removed and a number of approximately 50 cancer cell spheroids with a mean diameter in the range of 500 to 600 μm mixed with 200 μL collagen solution were placed in the dimple on the surface of the solidified collagen layer, followed by an additional 10 mins incubation period for gelation (Supplementary Fig. S1c,d). 1.8 mL collagen solution was then poured over the spheroids in the sample container (Supplementary Fig. S1e). The final mixture was incubated for complete gelation at a temperature of 37 °C for 1 hr (Supplementary Fig. S1f). After complete gelation, 0.5 mL of RPMI-1640 media was finally added into for preventing shrinkage of collagen gel. The collagen gel containing the cancer cell spheroids maintained at 37 °C in a humidified atmosphere with 5% CO_2_ until the HIFU exposure (Supplementary Fig. S1g). Detailed engineering drawings of the 3D printed cylindrical container and of the T-shaped bar used in the present study are provided in Supplementary Fig. S2.

### Viability and cytotoxicity assay

After boiling histotripsy exposure, 3D breast cancer cell spheroids embedded in the collagen gel were stained using Live/Dead Cell Staining Kit (BioVision, Cat#K501) to distinguish between live and dead cells. After incubation for 1 hour at 37 °C in a humidified atmosphere with 5% CO_2_, the embedded cells were fixed with 4% paraformaldehyde for overnight at 4 °C. Stained live and dead cells were visualised by confocal microscopy (Carl Zeiss, LSM700). Furthermore, the cell viability and cytotoxicity of boiling histotripsy were measured by GloMax® Discover Multimode Microplate Reader (Promega, Madison, WI, USA) using CellTiter-Glo® Luminescent Cell Viability Assay (Promega, Cat# G7570) and CellTox™ Green Cytotoxicity Assay (Promega, G8741).

### Cell death pathway and human macrophage polarisation markers with qPCR array

Total RNA in MDA-MB-231 cells was extracted using TRIzol reagent (Invitrogen, Cat#15596018). Briefly, one μg of total cellular RNA was converted to cDNA by RT² First Strand Kit for cDNA synthesis (Qiagen, Cat#330401). Real-time P**C**R was performed using diluted cDNA with the Human Cell Death Pathway Finder RT² Profiler PCR Array (Qiagen, Cat#330231) and GeneQuery™ Human Macrophage Polarization Markers qPCR Array (ScienCell, Cat#GK120) by the Applied Biosystems models 7500 real-time cyclers.

### Immunoblot

A total of 20 μg of protein from MDA-MB-231 cell extracts was analysed for immunoblotting and was detected by using primary antibodies specific for calreticulin (ab2907), HSP70 (ab181606), HMGB1 (ab18256) and β-Actin(sc-47778), which were purchased from Santa Cruz Biotechnology (Santa Cruz, CA, USA) and Abcam (Cambridge, MA, USA). For the immunoblotting, supernatants containing all the intracellular components from disrupted dead cells, and partially damaged cells as whole lysate after BH exposure were collected to analyse HMGB-1. Luminescent images were analysed using a luminoimager LAS-3000 (Fujifilm).

### Human cytokine array

Supernates obtained from boiling histotripsy-treated MDA-MB-231 cells were diluted and mixed with a human cytokine array biotinylated detection antibody cocktail (R&D Systems, Cat#ARY005B). The mixture was then incubated overnight with human cytokine array nitrocellulose membrane at 4 °C on a rocking platform. After washing to remove unbound materials, Streptavidin-HRP (horseradish peroxidase) and chemiluminescent detection reagents were added and incubated for 30 minutes at room temperature. Again, luminescent images were obtained using a luminoimager LAS-3000 (Fujifilm), and pixel densities on the developed membrane were analysed using ImageJ image analysis software.

### Human macrophage differentiation

THP-1 cells (2 × 10^5^ cells) were added into the six well plates per well with 50 ng/mL of 12-O-tetradecanoylphorbol-l3-acetate (PMA, Sigma, Cat#P1585) containing the 2 mL medium for 48 h. Activated THP-1 cells were differentiated to M1 or M2 macrophage by treating with 100 ng/mL of LPS (lipopolysaccharides) (Sigma, Cat#L2630) and 20 ng/mL of hIFN-ɤ (R&D systems, Cat#285-IF-100) in com RPMI or treating with 20 ng/mL of hIL-4 (R&D systems, #204-IL-010) and 20 ng/mL of hIL-13 (R&D systems, Cat#213-ILB-005) in com RPMI for 2 days.

### Flow cytometry analysis

THP-1 cells were seeded at a density of 2 × 10^4^ cells and were co-cultured with supernatant of boiling histotripsy treated MDA-MB-231 cells using 24 mm Transwell® with 0.4 µm pore polyester membrane insert (Corning, Cat#3450). For macrophage differentiation analysis, cells were blocked with human BD Fc Block™ (BD Bioscience, Cat#564219) and stained with APC anti-human CD197 (CCR7) Antibody (eBioscience™, Cat#14-1979-82) and PE anti-human CD209 (DC-SIGN) Antibody (BioLegend, Cat#330105). The assay was performed on a CytoFLEX flow cytometer (Beckman Coulter Life Sciences, Indianapolis, IN, USA) and was analysed using FlowJo_V10 software.

### Statistical analysis

Cell viability, cytotoxicity assay, cell death pathway qPCR array, cytokine array and macrophage polarisation qPCR array conducted in the present study were performed in triplicate. All statistical values were presented as mean ± standard deviation (SD). Student’s *t*-test was used to determine the statistical significance of results obtained in this study. A *p*-value of less than 0.05 was considered significant.

## Results

### Boiling histotripsy can cause breast cancer cell death

**Figure 4:**
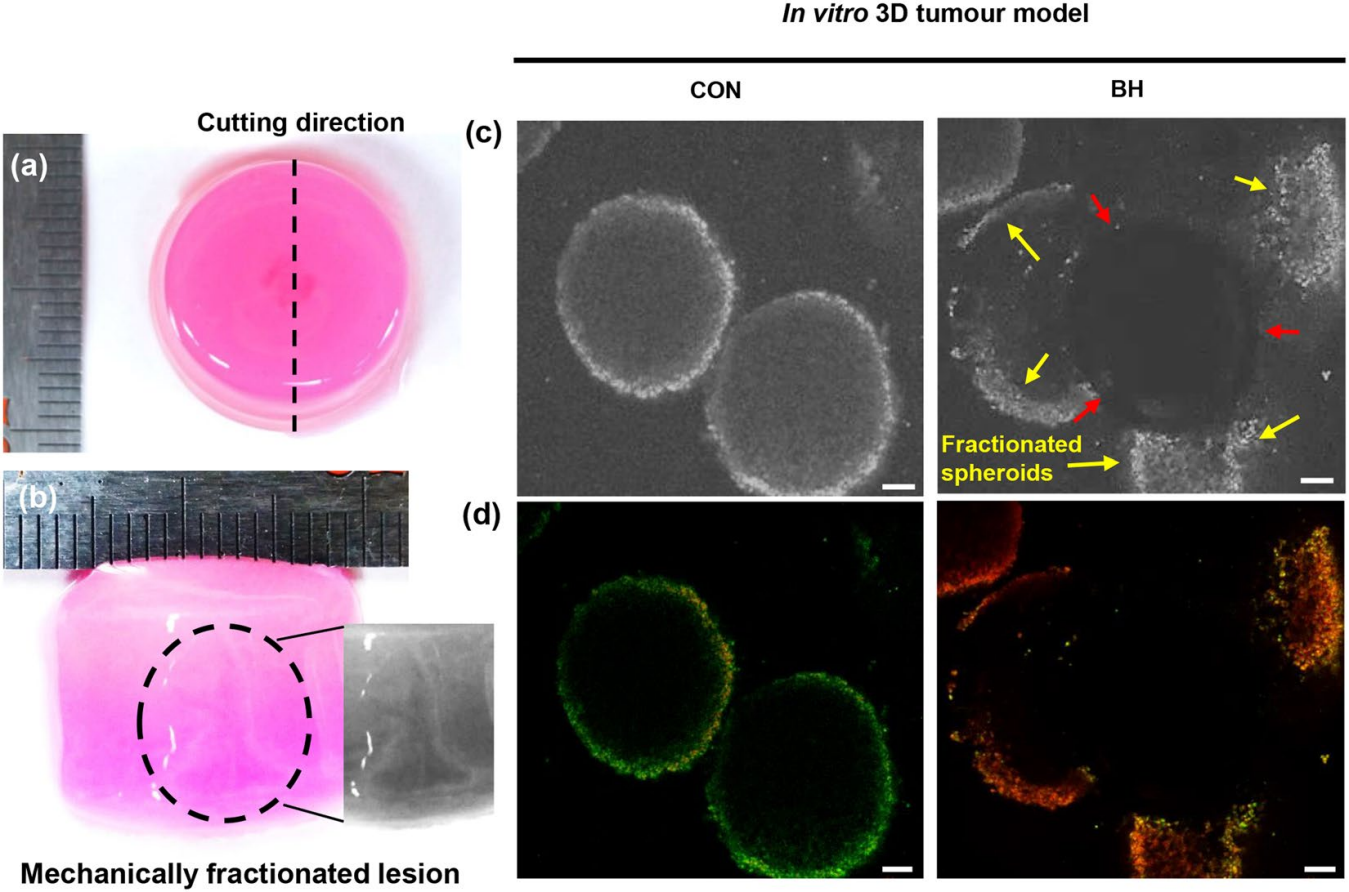
(**a**) Mechanical damage induced in the collagen gel in the absence of the 3D breast cancer cell spheroids at the HIFU focus after the boiling histotripsy exposure. Immediately after the HIFU exposure, the collagen gel was cut through the middle line (bottom view). (**b**) shows the cross-section of the collagen gel indicating a “tadpole” shaped mechanically fractionated lesion resulting from the boiling histotripsy exposure. The HIFU beam propagates from bottom to top. Inset: in greyscale. (**c**) Microscopic images of the observation of mechanically fractionated breast cancer cell spheroids in the 0.6% collagen gel. (**d**) Corresponding confocal images of (**c**) showing breast cancer cell death after the BH exposure. Live & Dead assay analysis was performed. A scale bar represents 100 μm. Five 10 ms HIFU pulses with *P*_+_ of 85 MPa, *P*_−_ of −14 MPa, duty cycle of 1% and PRF of 1 Hz were employed. CON = Control group (i.e., no boiling histotripsy exposure). BH = Boiling histotripsy.

Collagen is a major component of the extracellular matrix in various human tissues^25,26^. Therefore, many of the previous researchers have developed 3D tissue model using collagen and tried to utilise this physiologically relevant *in vitro* platform to precisely study cancer development and progression^27,28^. Figure 4a,b show photographs of the cross-sectioned collagen gel immediately after the five 10 ms boiling histotripsy pulses with *P*_+_ of 85 MPa and *P*_−_ of −14 MPa at the HIFU focus in the absence of the 3D breast cancer cell spheroids. This was performed to investigate whether mechanical damage can be induced in the collagen gel with the HIFU exposure conditions used in the present study. A well-defined “tadpole” shaped-mechanically fractionated lesion filled with liquid (i.e., liquefied lesion) was clearly observed at the HIFU focus in the gel (see Fig. 4b), which is a characteristic of boiling histotripsy exposure^22,29,30^.

After the confirmation of the generation of a boiling histotripsy lesion in the collagen gel, the 3D breast cancer spheroids embedded at the location of the HIFU focus in the gel were exposed to the same HIFU exposure conditions (i.e., five 10 ms HIFU pulses with *P*_+_ of 85 MPa, *P*_−_ of −14 MPa at focus, duty cycle of 1% and PRF of 1 Hz). Microscopic images depicted in Fig. 4c clearly show partial mechanical fractionation of 3D breast cancer spheroids at the HIFU focus in the collagen gel immediately after the boiling histotripsy exposure. Examining the spheroids morphology at the HIFU focal volume together with Live and Dead assay analysis, mechanical damage of the collagen gel was optically visible in the absence of the cell spheroids at the centre of the HIFU focal zone (indicated by red arrows in Fig. 4c) whereas partially fractionated cells (indicated by yellow arrows in Fig. 4c) appeared around this HIFU focal point. Since the degree of mechanical damage induced by BH is primarily dependent upon the number of BH pulses^11,22^, this partial mechanical fractionation shown in Fig. 4 was most likely to be due to the low number of BH pulses employed (i.e., five pulses). In addition, it was observed that most of these fractionated cells located on the outer surface of the spheroid were dead cells compared to those in the control group (i.e., no boiling histotripsy exposure) (Fig. 4d).

### Boiling histotripsy promotes immunogenic cell death of cancers and upregulates TNF-induced necrosis signalling

**Figure 5:**
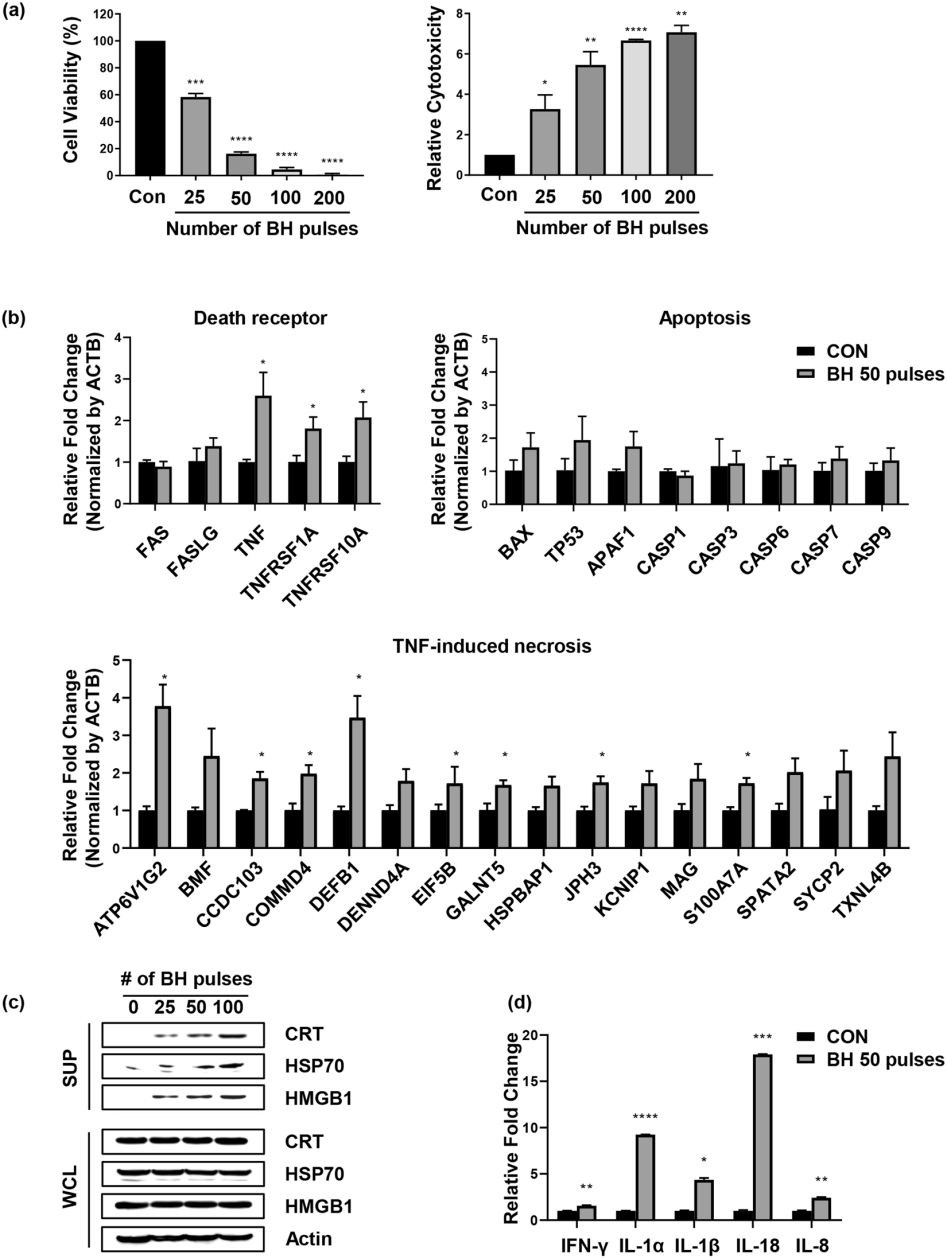
Immunogenic cell death induced by boiling histotripsy. (**a**) The changes of cell viability and cytotoxicity of boiling histotripsy with HIFU pulses are shown. Results of (**b**) cell death pathway analysis and (**c**) the secretion of damage-associated molecular patterns (DAMPs). (**d**) Cytokine array analysis showing the secretions of pro-inflammatory cytokines and chemokines from the breast cancer cells treated with boiling histotripsy. CON = Control group (i.e., no boiling histotripsy exposure). BH = Boiling histotripsy. *p ≤ 0.05, **p ≤ 0.01, ***p ≤ 0.001 and ****p ≤ 0.0001.

Since the boiling histotripsy used in the present study was able to cause the death of cancer cells, additional experiments were carried out to examine (a) the relationship between cell viability and the degree of mechanical damage, and (b) the underlying mechanism associated with cancer cell death resulting from the boiling histotripsy exposure. For the experiment, the cancer cells were exposed to a number of boiling histotripsy pulses which varied from 25 to 200 pulses whilst other HIFU exposure conditions including the peak acoustic pressures at the HIFU focus, pulse length, duty cycle, and the pulse repetition frequency were kept constant. Figure 5a shows a gradual reduction in cell viability as well as an increase in cytotoxicity with boiling histotripsy pulses.

To understand the underlying mechanisms related to the cell death induced by the boiling histotripsy, cell death pathway qPCR array analysis was also conducted an hour after the boiling histotripsy (BH) exposure. Supernatants of breast cancer cells treated with 50 BH pulses were used for this assay. With this exposure condition, partially damaged cells appeared (i.e., partial BH treatment, see Fig. 5a), and therefore, the qPCR analyses performed were limited to partial mechanical fractionation only. Results interestingly indicated that: (1) similar levels of expression of FAS and FASLG genes were observed between the control and the BH-treated groups; (2) the levels of TNF (Tumour Necrosis Factor) signalling pathway markers in the BH treated group were significantly higher than those of the control group; (3) TNF-induced necrosis markers were highly expressed by the BH exposure, and (4) there were no significant differences in the caspases between the BH treated and the control groups (Fig. 5b). Furthermore, danger signals such as calreticulin (CRT), heat-shock proteins (HSPs), high mobility-group box 1 (HMGB-1) were observed in the BH-treated group which gradually increased with the number of BH pulses. In contrast, CRT, HSPs and HMGB-1 were hardly detected when MDA-MB-231 cells were exposed to cisplatin or doxorubicin (apoptosis-inducing chemicals) (Supplementary Fig. S3a). Along with the observations of damage- or danger-associated molecular patterns (DAMPs) under the boiling histotripsy exposure, pro-inflammatory cytokines such as interferon (IFN)-g and interleukin (IL)-1 family and chemokines (IL-8), which are typically involved in recruiting immune cells, were observed in the BH-treated group (Fig. 5d and Supplementary Fig. S3b). On the basis of the results above, we concluded that mechanical stresses produced by boiling histotripsy can induce immunogenic cell death of cancers via TNF-mediated necrosis signalling pathway.

### Boiling histotripsy may stimulate M1 macrophage polarisation

**Figure 6:**
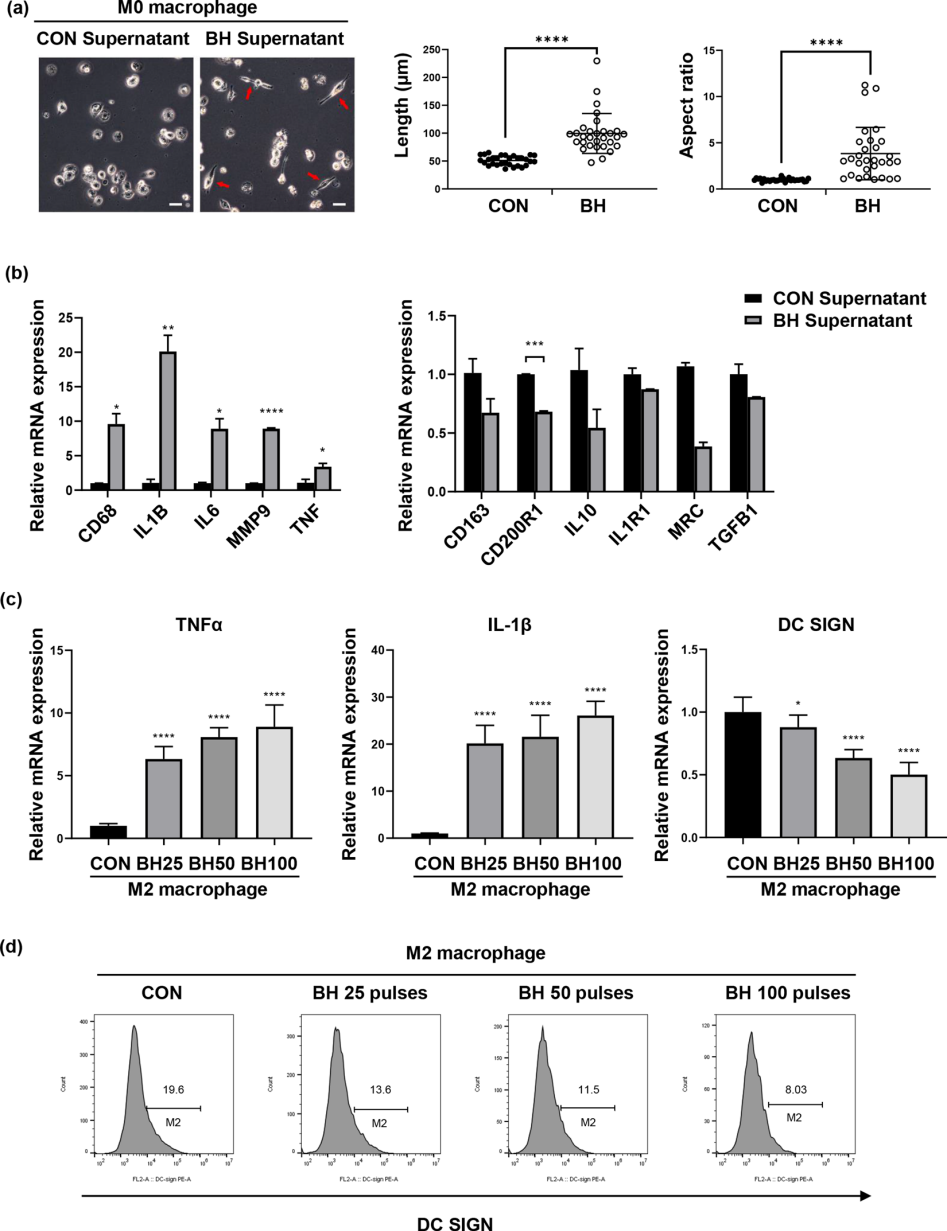
Boiling histotripsy-induced breast cancer cell death stimulates M1 macrophage polarisation. (**a**) The morphological changes of macrophages from M0 to M1 like cells after co-cultured with the supernatant of the boiling histotripsy-treated breast cancer cells. A scale bar represents 100 μm. (**b**) Increases of M1 macrophage-related markers and decreases of M2 macrophage-related markers are shown. 50 boiling histotripsy pulses were used. (**c**,**d**) Reprogramed macrophages, from M2 to M1 like, after cultured with the supernatant obtained from the boiling histotripsy-treated cancer cells. CON = Control group (i.e., no boiling histotripsy exposure). BH = Boiling histotripsy. *p ≤ 0.05, **p ≤ 0.01, ***p ≤ 0.001 and ****p ≤ 0.0001.

Since immunogenic cell death induced by boiling histotripsy secreted various pro-inflammatory cytokines (Fig. 5c,d), we hypothesised that boiling histotripsy may also modulate a tumour microenvironment (TME). To test this hypothesis, macrophage polarisation assay was performed. The morphological changes of M0 macrophages co-cultured with the supernatant of the boiling histotripsy-treated breast cancer cells are shown in Fig. 6a. In general, M0 macrophage derived from THP-1 cells has a round shape whereas M1 macrophage-like cells can be observed as elongated cells, like a spear^31^. A number of M1 macrophage-like cells were optically observed under a microscope (indicated by the red arrows in Fig. 6a). The length as well as **t**he aspect ratio of the M1 like cells increased compared to those in the control group. To further investigate this, macrophage polarisation markers (i.e., TNF-alpha and IL-1B markers for M1 and DC SIGN marker for M2) were analysed with qPCR. Interestingly, M1 macrophage markers significantly increased with reduced levels of the M2 macrophage markers after M0 macrophage was cultured with the supernatant obtained from the boiling histotripsy-treated cancer cells (Fig. 6b). In addition to the qPCR results, flow cytometry analyses also showed that the supernatant from the boiling histotripsy-treated cancer cells was able to induce an increase of M1 macrophage markers (Fig. 6c), but a decrease of M2 macrophage markers (Fig. 6c,d and Supplementary Fig. S4).

## Discussion

In recent years, significant interest has been garnered in potential clinical applications of boiling histotripsy due to its capability of mechanically fractionating target tissue without causing any significant thermal damage as well as without leaving scar tissue behind. In comparison to traditional HIFU thermal ablation, boiling histotripsy can create a sharper transition zone between treated (completely destructed tissue) and untreated regions (completely intact) with a transition distance of less than a cell length (of the order of a micrometre). This is possible because boiling histotripsy treatment is not greatly affected by heat perfusion via blood flow^18,32^, thus enabling precise targeting of tumour volumes and sparing of surrounding normal healthy tissue. During HIFU thermal ablation, for instance, blood perfusion can result in undertreating a targeted tissue and surrounding tissue can possibly be thermally ablated due to thermal diffusion^2,4^. Furthermore, tissue debris remaining inside a boiling histotripsy-induced lesion is likely to be absorbed as part of the physiologic healing response, whereas HIFU-induced thermal lesion becomes fibrous scar tissue^33^.

With a growing interest in immunotherapy for cancer treatment, a study of the HIFU-induced mechanical effects on anti-tumour immunity recently becomes one of the most attractive research areas in the HIFU fields^18,34^. In the present study, we extensively investigated (a) the feasibility of using boiling histotripsy to mechanically destroy human breast cancer cells (MDA-MB-231), (b) the potential immunological effects of boiling histotripsy and (c) the mechanisms underlying the immune response to boiling histotripsy, particularly the intercellular process of the generation of pro-inflammatory cytokines as well as macrophage polarisation. Furthermore, the relationship between the variation of the level of mechanical damage induced by boiling histotripsy and the corresponding immune activity was also examined *in vitro*.

Studies have reported that the shape of a boiling histotripsy lesion produced in soft tissue is “tadpole” like consisting of a “head” and a “tail” with the “head” toward the HIFU transducer^7,22^. We also observed a well-defined tadpole-shaped mechanically fractionated lesion in the collagen gel, as shown in Fig. 4b. This lesion shape formation is likely to be due to the number of boiling vapour bubbles induced at the HIFU focus and the violent inertial cavitation clouds formed in between the HIFU source and the boiling bubble during the HIFU exposure^22,24^. The generation of a nonlinear shocked wavefront at the HIFU focus due to nonlinear wave propagation effects in soft tissue can dramatically increase tissue temperature to boiling temperature in a few milliseconds. A boiling vapour bubble subsequently forms within this localised heated volume and grows to millimetre size due to the combination of asymmetric in a shockwave and water vapour transport (i.e., rectified bubble growth)^23,24,35^. Further interaction between incoming incident shockwaves and the acoustic fields backscattered by the boiling bubble can result in the formation of a cavitation cluster in front of the bubble progressing toward the HIFU source (Supplementary Fig. S5a)^22,36^. Shear stresses produced around an oscillating a boiling bubble at the HIFU focus may cause mechanical tissue damage^24^, whereas emissions of micro-jetting and shockwaves resulting from a cavitation cluster enable the mechanical disruption of tissue (Supplementary Fig. S5b)^7,22,30,32^. These mechanical stresses created by boiling bubbles and cavitation clouds can cause mechanical damage to human breast cancer cells, eventually leading to cell death (Fig. 4c,d). It is worth mentioning that the BH exposure conditions used in the present *in vitro* study resulted in partial cell damage. In *in vivo* situation, this type of partial fractionation would likely be observed at the margin of a BH lesion.

After the confirmation of boiling histotripsy-induced mechanical damage of human breast cancer cells, we further investigated the types of cell death caused by boiling histotripsy. Our experimental results indicated that the levels of the markers expression of FAS signalling pathway (FAS and FASLG) and of the caspases (−1, −3, −6, −7 and −9) were not changed, whereas TNF signalling pathway markers (TNF, TNFRSF1A and TNFRSF10A) significantly increased after the boiling histotripsy exposure (Fig. 5b). Since the stimulation of death receptor signalling can trigger TNF-induced necrosis in the absence of caspase activation^37,38^, the main cause of cell death by boiling histotripsy is therefore likely TNF-induced necrosis rather than apoptosis.

Immunogenic cell death is characterised by the presence of specific molecules including calreticulin (CRT), heat-shock proteins (HSP70 and HSP90) and high mobility-group box 1 (HMGB-1). These protein molecules are known as damage- or danger-associated molecular patterns (DAMPs) and typically function as an activation of “eat me” signal for professional phagocytes such as macrophage, which can lead to immunostimulation through presentation of tumour antigens to antigen-presenting cells (APCs)^39,40^. We observed the release of DAMPs (CRT, HSP 70, HMGB-1) after the boiling histotripsy exposure (Fig. 5c,d). Similar results have also been reported in other HIFU studies^41–45^, where a rapid release of DAMPs was observed following HIFU thermal ablation. Though DAMPs can also be released by HIFU thermal ablation, studies^16,42^ suggest that mechanically lysed tumour cells by HIFU may release larger amounts of tumour antigens and DAMPs at the HIFU focus *in situ*, which are not thermally denatured. Kramer *et al*.^41^ experimentally observed the highest upregulation of HSP70 at the border zone of the HIFU-induced thermally ablated lesion in prostate specimens *in vivo*. Further studies on quantitative and qualitative comparisons of the types of secreted tumour antigens and DAMPs after HIFU thermal ablation or boiling histotripsy are necessary.

In this study, in addition to the release of DAMPs, significant increases in the secretions of pro-inflammatory cytokines (IFN-γ, IL-1α, IL-1β and IL-18) and of chemokines (IL-8), which recruit and activate immune cells, were observed after the boiling histotripsy exposure (Fig. 5d). This is of paramount importance for immunotherapy as these particular cytokines and chemokines generally activate macrophages to M1 macrophages (Fig. 6a,b) which have pro-inflammatory, bactericidal, phagocytic and anti-tumour functions. Macrophages play an important role in the growth or regression of tumours. Specifically, tumour associated macrophages (TAMs) are the main populations of tumour-promoting inflammatory cells which are mainly polarised M2-like macrophages^46^. TAMs contribute to creating an immunosuppressive tumour microenvironment (TME) which produces growth-promoting molecules that actively stimulate tumour growth^47^. One of the recent immunotherapeutic strategies for cancer therapy is, therefore, the functional reprogramming of TAMs to an anti-tumour, ‘M1-like’ phenotype^48,49^. In this context, our results suggest that boiling histotripsy could potentially be used to modulate a tumour immune microenvironment by reprogramming of M2 to M1 like-macrophages (Fig. 6d). Further detailed investigations are warranted in this aspect.

Our *in vitro* results together with a recent *in vivo* work reported in Schade *et al*.^20^ clearly demonstrated the potential immunological effects of boiling histotripsy. On the basis of the results presented in this work (i.e., partial mechanical fractionation after BH) with the help of Matzinger’s danger theory^50^, we speculate the potential mechanisms of boiling histotripsy-induced immunomodulation as follows. Mechanical stresses produced by acoustic cavitation which occurs during boiling histotripsy can cause immunogenic cell death via TNF-induced necrosis. Tumour antigens and DAMPs such as CRT, HSP70, HSP90, and HMGB-1 are likely to be released from boiling histotripsy-treated tumour cells at the HIFU focus *in situ*, followed by the generations of pro-inflammatory cytokines and chemokines. These signaling proteins can then activate macrophages to M1 macrophages, thus modulating tumour microenvironment and triggering an immune system against tumour cells. The desired level of immune response may be achieved by varying the degree of mechanical damage induced by boiling histotripsy (Fig. 5a,c).

Despite the recent progress in the development of HIFU techniques, local recurrence of the tumour, residual tumour cells at the treatment site and emergence of metastases are currently the main potential clinical problems associated with HIFU treatment^34,51^. To improve the clinical outcomes, combination therapies such as HIFU thermal or HIFU mechanical ablation (e.g., boiling histotripsy) with immune checkpoint immunotherapy would be necessary and warrant further investigation. Preclinical studies investigating the potential synergetic effects of boiling histotripsy and programmed cell death-1 receptor (PD-1) and its ligand PD-L1 pathway blockade therapy for the treatment of breast cancer are currently ongoing.

## Conclusions

In this paper, we clearly demonstrated the feasibility of using and the immunological effects of boiling histotripsy for the treatment of human breast cancer. After the boiling histotripsy exposure, cancer cells were mechanically fractionated and underwent cell death via TNF-induced necrosis. Along with the observation of the cell death, significant increases in secretions of DAMPs, pro-inflammatory cytokines, and chemokines that were related to M1 macrophage activation appeared. Together, our results can suggest that boiling histotripsy might be an invaluable tool for immunotherapy as not only it can mechanically destroy solid tumours (e.g., breast cancer) but also can induce immunogenic cell death to trigger antitumour immunity.

## Supplementary information

Supplementary Materials
