# Supplementary material for: Boiling Histotripsy-induced Partial Mechanical Ablation Modulates Tumour Microenvironment by Promoting Immunogenic Cell Death of Cancers
Source: Sci Rep. 2019 Jun 21;9:9050. doi: 10.1038/s41598-019-45542-z (PMC6588624; doi:10.1038/s41598-019-45542-z)

**Supplementary Materials**

**Title:** Boiling Histotripsy-induced Partial Mechanical Ablation modulates Tumour Microenvironment by Promoting Immunogenic Cell Death of Cancers

**Authors and affiliations:** Ki Joo Pahk^1,*^, Cheol-Hee Shin^2,*^, In Yeong Bae^2^, Yoosoo Yang^3^, Sang-Heon Kim^2^, Kisoo Pahk^4,5^, Hyungmin Kim^1,#^, and Seung Ja Oh^2,#^

^1^Center for Bionics, Biomedical Research Institute, Korea Institute of Science and Technology (KIST), Seoul, 02792, Republic of Korea

^2^Center for Biomaterials, Biomedical Research Institute, Korea Institute of Science and Technology (KIST), Seoul, 02792, Republic of Korea

^3^Center for Theragnosis, Biomedical Research Institute, Korea Institute of Science and Technology (KIST), Seoul, 02792, Republic of Korea

^4^Institute for Inflammation Control, Korea University, Seoul, 02841, Republic of Korea

^5^Department of Nuclear Medicine, Korea University Anam Hospital, Seoul, 02841, Republic of Korea

^*^These authors contributed equally to this study.

^#^Co-corresponding authors.

**Running title:** Boiling histotripsy for the treatment of human breast cancer

**Keywords:** High Intensity Focused Ultrasound, Boiling Histotripsy, Human Breast Cancer Cells, Immunogenic Cell Death, Tumour Microenvironment

**Financial support:** This work was supported by the National Research Council of Science & Technology (NST) grant by the Korea government (MSIT) (No. CAP-18-01-KIST) and KIST Institutional Program (2E27975 & 2E29340).

**Conflict of interest:** The authors declare no potential conflicts of interest.

**Corresponding authors:**

Hyungmin Kim^1^ and Seung Ja Oh^2^

^1^Center for Bionics, Biomedical Research Institute, Korea Institute of Science and Technology (KIST), Seoul, 02792, Republic of Korea. Phone: +82(0)29585695; Fax: +82(0)29585629; E-mail: hk@kist.re.kr

^2^Center for Biomaterials, Biomedical Research Institute, Korea Institute of Science and Technology (KIST), Seoul, 02792, Republic of Korea. Phone: +82(0)29585353; Fax:+82(0)29585308; E-mail: [seungja.oh@kist.re.kr](mailto:seungja.oh@kist.re.kr)

**Supplementary Figures**


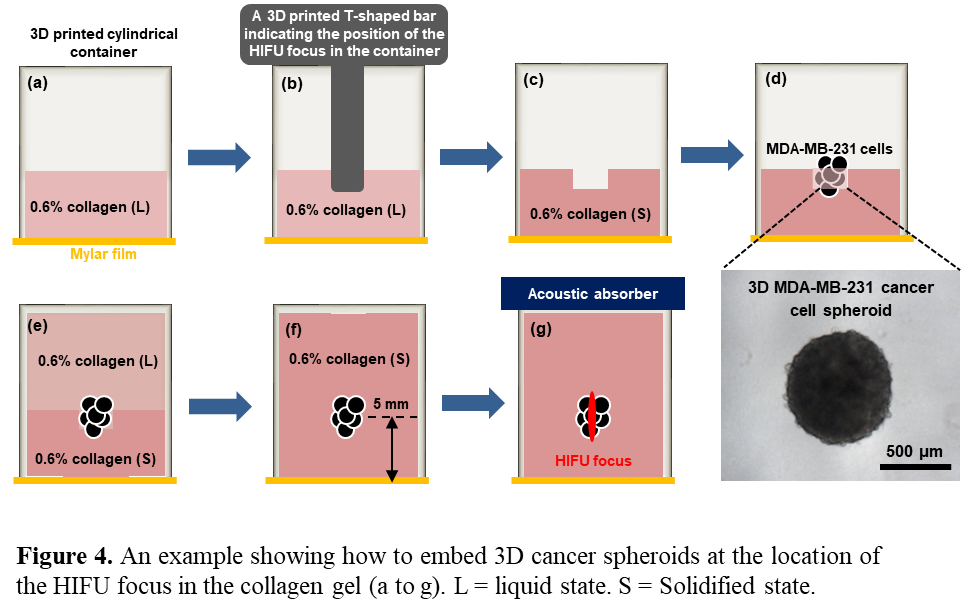


Supplementary figure S1. Preparation of 3D Tumor Model for boiling histotripsy exposure. An example showing how to embed cell spheroids at the location of the HIFU focus in the gel (a to g). L = liquid state. S = Solidified state.

**
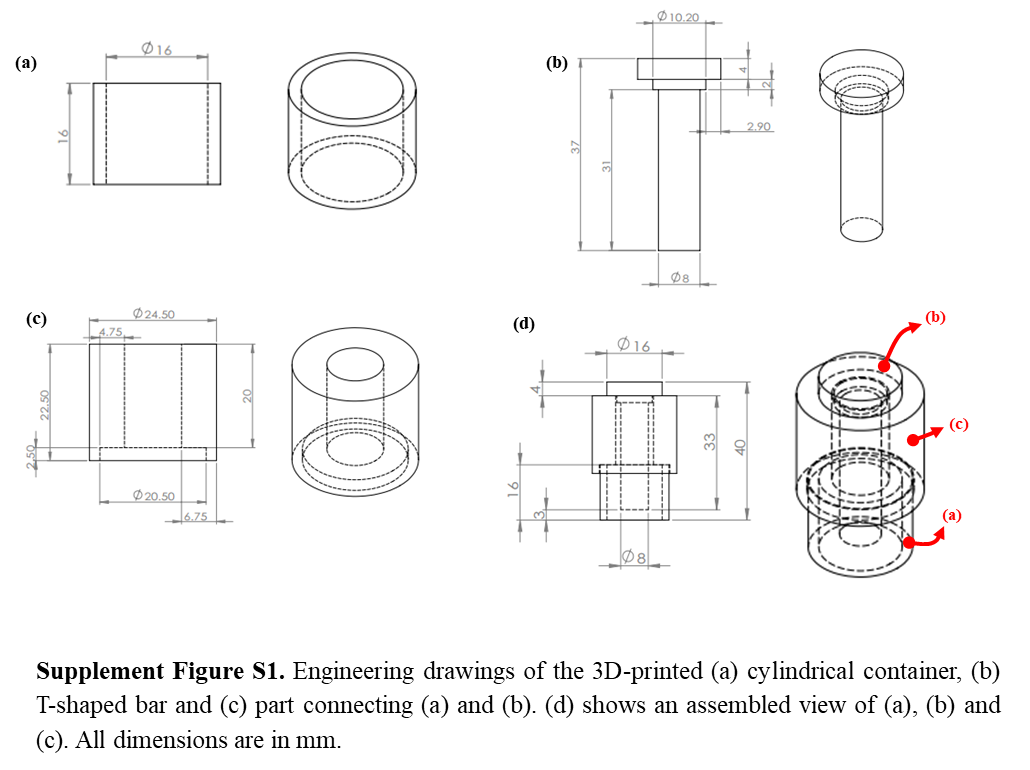
**

**Supplementary figure S2.** Engineering drawings of the 3D-printed (a) cylindrical container, (b) T-shaped bar and (c) the part connecting (a) and (b). (d) shows an assembled view of (a), (b) and (c). All dimensions are in mm.

**
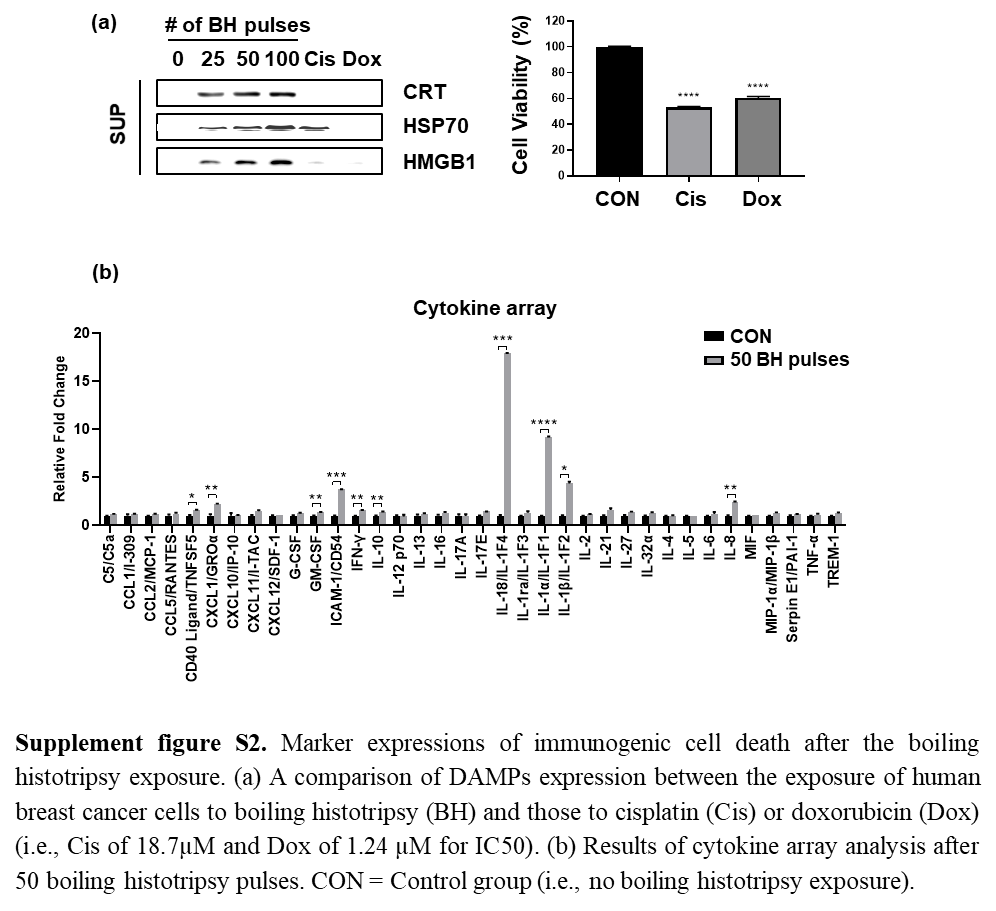
**

**Supplementary figure S3.** Marker expressions of immunogenic cell death after the boiling histotripsy exposure. (a) A comparison of DAMPs expression between the exposure of human breast cancer cells to boiling histotripsy (BH) and those to cisplatin (Cis) or doxorubicin (Dox) (i.e., Cis of 18.7µM and Dox of 1.24 µM for IC50). (b) Results of cytokine array analysis after 50 boiling histotripsy pulses. CON = Control group (i.e., no boiling histotripsy exposure). * = *p* ≤ 0.05, ** = *p* ≤ 0.01, *** = *p* ≤ 0.001 and **** = *p* ≤ 0.0001.

**
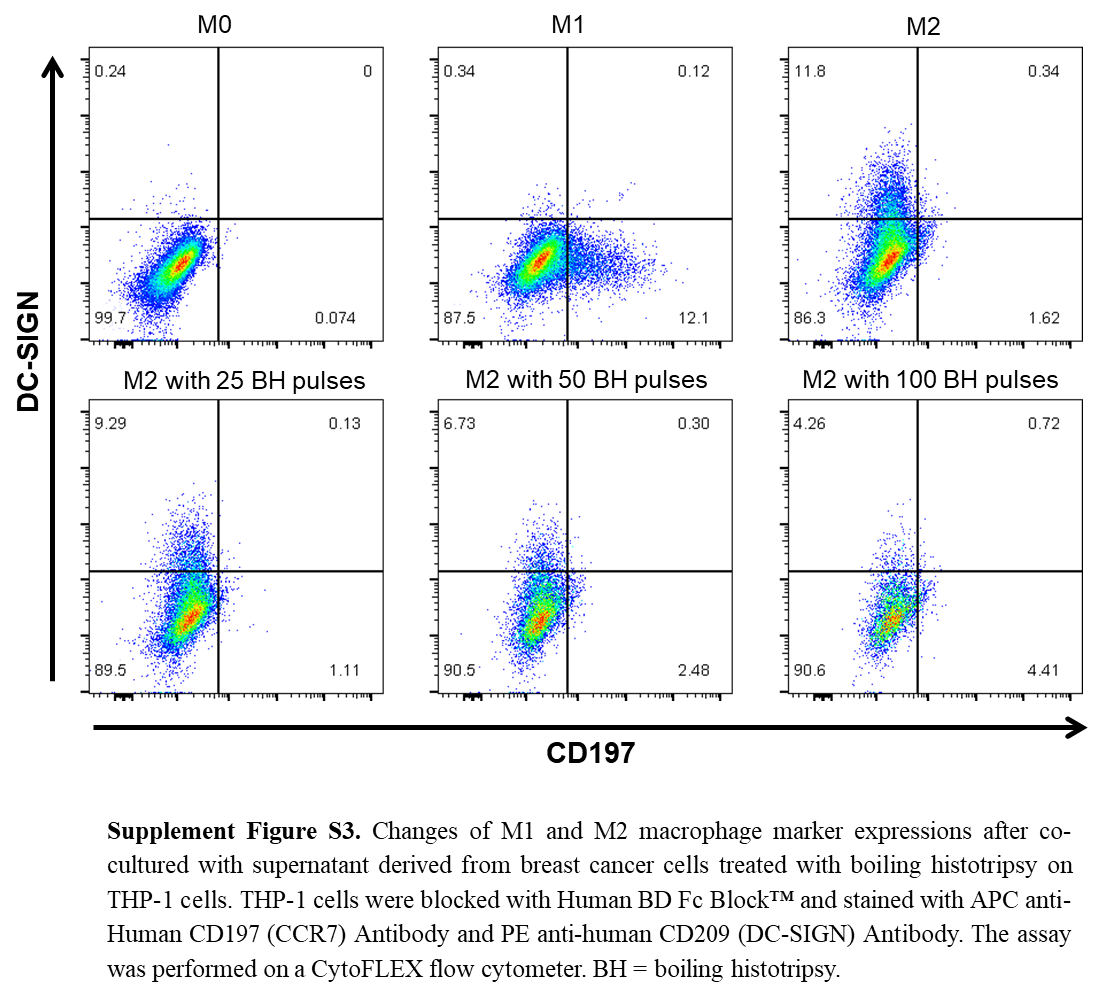
**

**Supplement figure S4.** Changes of M1 and M2 macrophage marker expressions after THP-1 cells were co-cultured with the supernatant of the boiling histotripsy-treated breast cancer cells. THP-1 cells were blocked with Human BD Fc Block™ and stained with APC anti-Human CD197 (CCR7) Antibody and PE anti-human CD209 (DC-SIGN) Antibody. The assay was performed on a CytoFLEX flow cytometer. BH = boiling histotripsy.

**
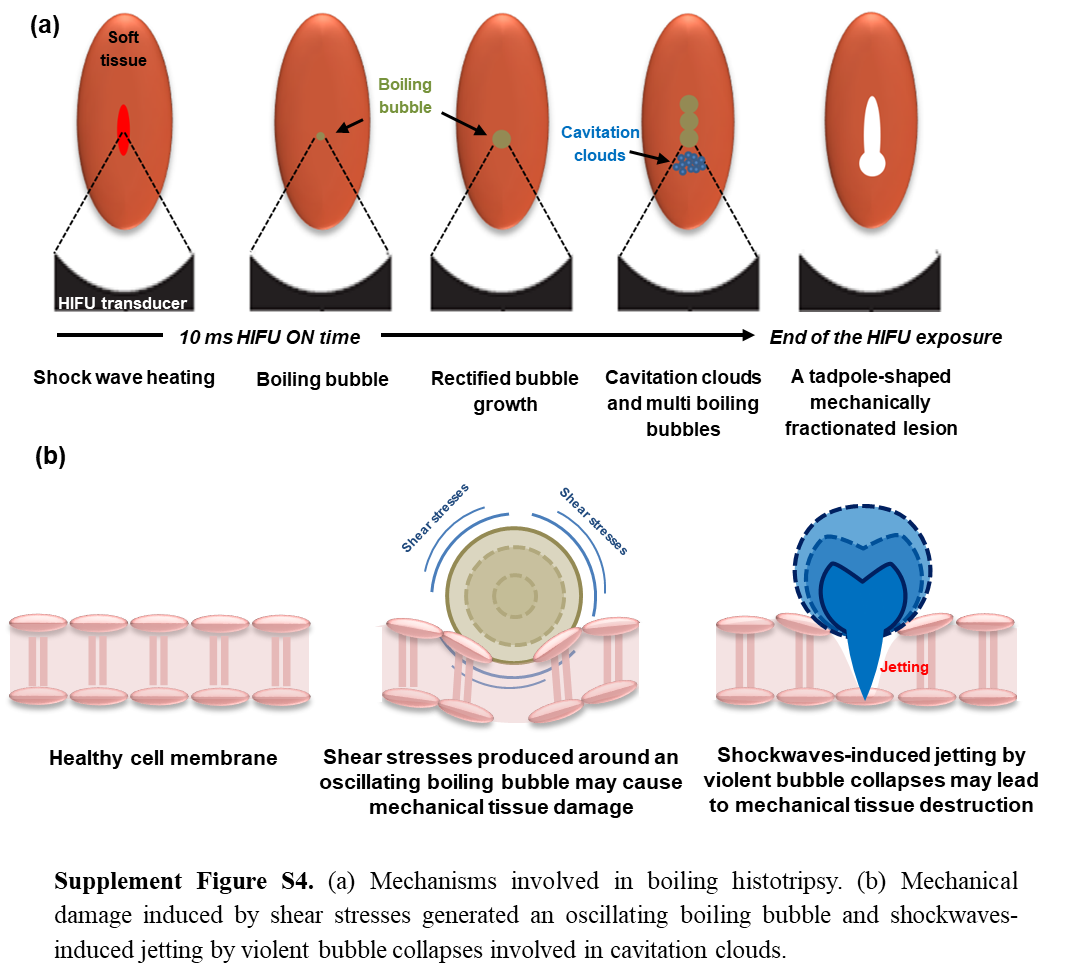
**

**Supplement figure S5.** (a) Mechanisms involved in boiling histotripsy [22]. (b) Mechanical damage induced by shear stresses generated around an oscillating boiling bubble and shockwaves-induced jetting by violent bubble collapses involved in cavitation clouds.

**Supplementary Table**

Table S1. Acoustic properties of the 0.6% collagen gel used in the KZK simulation. Because the main ingredient of the collagen gel was water, the acoustic properties of water [10] were used to approximate those of the 0.6% collagen gel used.

| **Acoustic properties** | **Value** |
| --- | --- |
| Mass density | 1000 kg/m^3^ |
| Speed of sound | 1481 m/s |
| Attenuation at 1 MHz | 0.217 dB/m |
| Coefficient of nonlinearity | 3.5 |

**Figure 5.** (a)

**Figure 5.** (b)

Death receptor

Apoptosis

TNF-induced necrosis

**Figure 5.** (c)

**WCL**

**SUP**

**0 25 50 100 BH Pulses**

**Actin**

**HMGB1**

**HSP70**

**CRT**

**HMGB1**

**HSP70**

**CRT**

**Figure 5.** (d)

**Figure 6.** (a)

**Figure 6.** (b)

M1 markers

M2 markers

**Figure 6.** (c)

**Supplementary figure S3.** (a)

**SUP**

**0 25 50 100 Cis Dox**

**HIFU Pulses**

**HMGB1**

**HSP70**

**CRT**

**Supplementary figure S3.** (a)


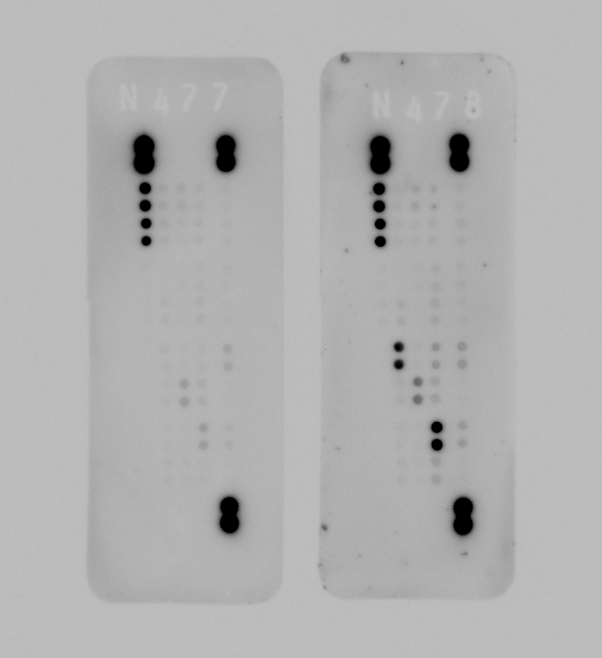

Supplement: Supplementary file 1 — Supplementary Materials [file 41598_2019_45542_MOESM1_ESM.docx]
